# Moderate to advanced periodontitis contributes to increased oxidative stress in cats: a case-control study

**DOI:** 10.1186/s12917-024-04110-y

**Published:** 2024-06-07

**Authors:** Hamidreza Moosavian, Marzie Gholikhani, Iraj Ashrafi Tamai, Mahsa Fazli

**Affiliations:** 1 Department of Clinical Pathology, Faculty of Veterinary Medicine, University of Tehran, Tehran, Iran; 2 Department of Microbiology and Immunology, Faculty of Veterinary Medicine, University of Tehran, Tehran, Iran; 3 Department of Biology, Faculty of Basic Science, Islamic Azad University, Tehran, Iran

**Keywords:** Periodontitis, Gingivitis, Oxidative stress, Cat

## Abstract

**Background:**

Periodontal diseases are the most frequently diagnosed problem in cats. It has been well-established that periodontal diseases could not only cause various oral health issues but could also contribute to systemic diseases. Oxidative stress is a possible link between systemic diseases and periodontitis. Our study aimed to illustrate the influence of periodontitis on oxidative stress development in cats. Furthermore, the changes in the bacterial flora of the gums were investigated.

**Methods:**

Based on the clinical and laboratory examinations, fifty cats were divided into two groups normal (*n* = 25) and moderate to advanced periodontitis (*n* = 25). Serum total antioxidant capacity (TAC), total oxidant status (TOS), reduced (GSH) and oxidized glutathione (GSSG) were measured. In addition, samples were taken from the subgingival plaques of all cats for bacterial culture.

**Results:**

Serum TOS, GSSG, GSSG to GSH ratio, and oxidative stress index (OSI), calculated as the ratio of TOS to TAC in cats with periodontal disease were significantly higher, and TAC was significantly lower (*p* < 0.05) compared with controls. The results of bacterial culture indicated that the number of isolated bacterial colonies is higher in patients than in the control group. Additionally, the analysis of these data showed a positive association between periodontal index and oxidative stress.

**Conclusions:**

Our results revealed that periodontitis in cats is related to a main oxidative stress. Furthermore, oxidant factors such as TOS and OSI, compared to antioxidant factors, may better indicate the presence of oxidative stress conditions in patients with periodontitis.

## Background

Periodontal diseases are highly prevalent in cats, posing significant challenges in terms of treatment and prevention. In the United States, periodontal disease is the most commonly diagnosed condition in cats, with calculus and gingivitis reported in approximately 24% and 13% of all cats, respectively [1]. This disease leads to various oral health issues, including gingivitis, loss of tooth-supporting structures, tooth mobility, and pain [2]. Timely and proper oral healthcare can reverse the initial stages of gingivitis. However, if left untreated, gingivitis can progress to periodontitis, which is difficult to resolve and may result in tooth loss. Periodontal disease can have local effects such as oronasal fistulas, perio-endo lesions, pathologic fractures, ocular problems, osteomyelitis, and a higher incidence of oral cancer [2].

Furthermore, periodontal disease is known to have systemic effects. Studies in humans have shown that individuals with generalized or localized periodontitis exhibit elevated levels of circulating leukocytes, serum C-reactive protein, and various cytokines including IFN-γ, TNF-α, IL-1β, IL-2, and IL-6, as well as IgG1 and IgG2, compared to control subjects [3, 4]. There is also a recognized association between periodontal disease and myocardial infarction in these patients [3]. In dogs, periodontal disease has been correlated with degenerative and inflammatory conditions in the liver, kidney, and left atrioventricular heart valves [5]. Studies conducted on dogs have also indicated that periodontal diseases are a risk factor for endocarditis [6]. In a recent study involving cats, a direct correlation was found between the severity of periodontitis and various parameters such as total globulins, ALT activity, IgG levels, anemia, and hypoalbuminemia [7]. Moreover, periodontitis has been identified as a significant risk factor for the development of impaired renal function and chronic kidney diseases, which are common issues in mature and older cats [8].

Periodontitis is an inflammatory condition that occurs in response to plaque biofilm and is characterized by inflammation and destruction of the supporting tissues around the teeth [9]. This process involves the excessive production of reactive oxygen species, making oxidative stress a pivotal factor in the pathogenesis of both local and systemic disorders [9]. Oxidative stress arises when there is an imbalance between the production and accumulation of reactive oxygen species in cells and tissues. Recently, oxidative stress has been proposed as a central link between systemic diseases and periodontitis in humans [10].

There are a wide range of oxidant and antioxidant factors available in the body that contribute to the overall oxidative balance. These factors include individual nonenzymatic antioxidants (such as vitamin C, vitamin E, glutathione and flavonoids), enzymes involved in antioxidant defense, and various oxidant molecules [11]. It is important to note that the changes in these parameters may not always occur in a uniform manner in different disorders or conditions. Rather than focusing on individual antioxidants or oxidants, total antioxidant capacity (TAC) and total oxidant status (TOS) take into account the cumulative effects and interactions of multiple components involved in the oxidative balance, and so play a crucial role in understanding the oxidative balance in the body [12]. Higher TAC values indicate a great ability to neutralize ROS and maintain oxidative balance, and elevated TOS levels indicate increased oxidative stress and a greater potential for tissue damage [13]. Furthermore, oxidative stress index (OSI), calculated as the ratio of TOS to TAS, is considered a more accurate indicator of oxidative stress [12].

Among various types of antioxidant factors, glutathione, often referred to the master antioxidant, plays a primary mechanism against oxidative stress in all tissue especially periodontal tissue [14–16]. It helps to protect oral cells from oxidative damage caused by the excess production of ROS during inflammation [15, 16].

Despite the high incidence and significance of periodontal diseases in cats, there is a lack of information regarding the role of oxidative stress in cats affected by periodontitis. The present study aimed to evaluate the presence and severity of oxidative stress in cats with moderate to severe periodontitis.

## Methods

### Study design and patient selection

We performed a case-control study of 50 cats presented to the Veterinary Teaching Hospital of the University of Tehran between 2022 and 2023 and underwent a complete physical examination, laboratory survey, and abdominal ultrasonography on the same day.

All methods are reported in accordance with ARRIVE guidelines (https://ariveguidelines.org) for the reporting of animal experiments. All phases of this study were approved by the Animal Ethical Guidelines of the Faculty of Veterinary Medicine, University of Tehran, Tehran, Iran (No: 8509019.7.44).

**Fig. 1:**
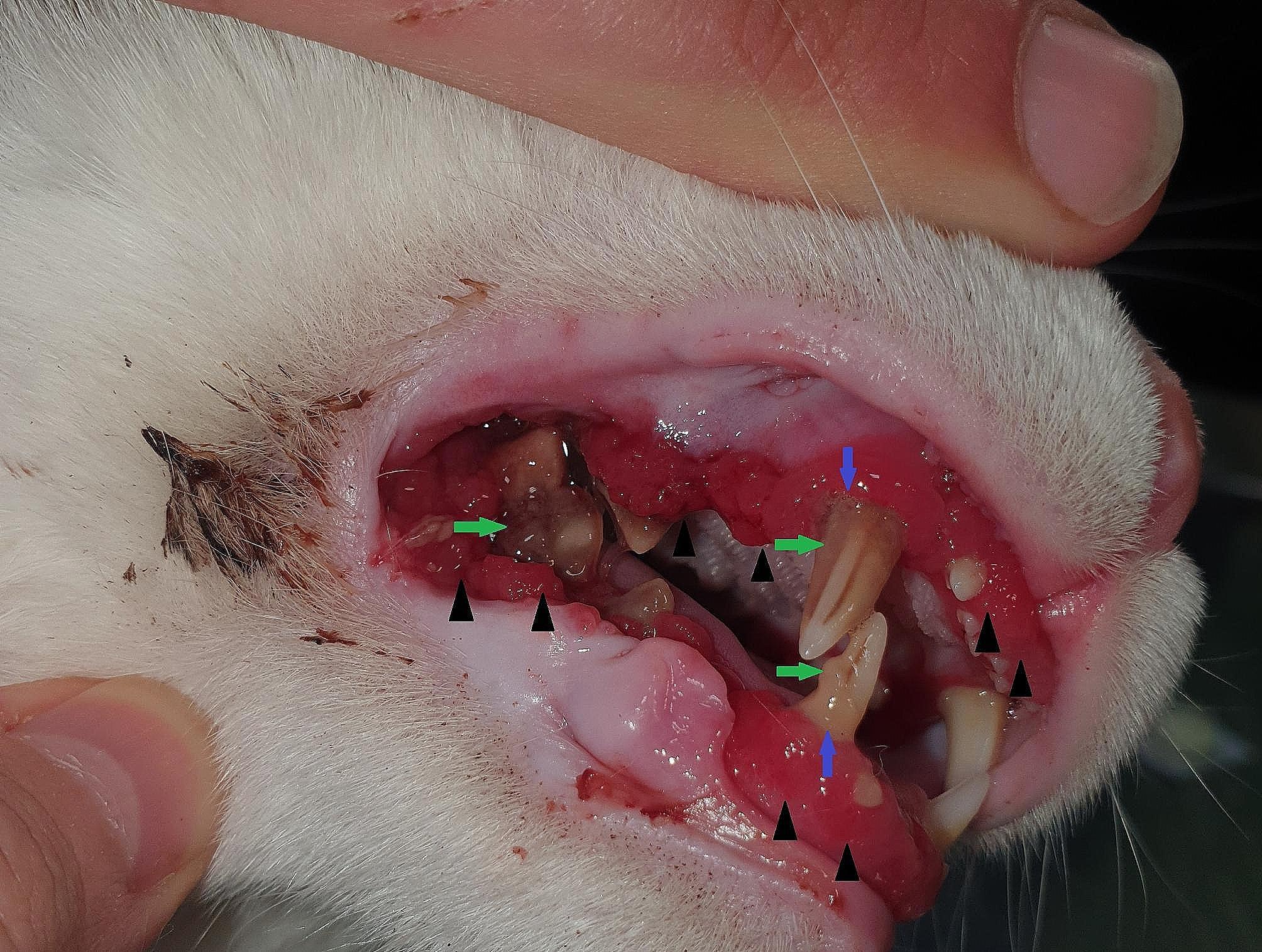
Advanced periodontitis in a 12-month-old cat. Severe periodontitis as gingival inflammation and hyperplasia (black arrowhead), abundant plaque on the teeth (green arrows), gingival recession (blue arrow), and the formation of the periodontal pocket that is visible around the upper canine tooth

For the study, approximately 300 cats from 6 months to 3 years were examined initially, and cats were selected for inclusion in the study based on moderate to advanced periodontitis confirmation (Fig. 1). For clinical periodontal examinations, all evaluations were performed by the same clinicians.

Periodontal probing depth (PP) and gingival recession (R) were measured to calculate the periodontal (or clinical) attachment loss (PAL) by the following formula: PAL = PP ± *R* + 0.5 mm. A 5-point scale for grading the periodontal index was used [17]. Briefly, Stage 0: Clinically normal; Stage 1: Gingivitis only without attachment loss; Stage 2: Early periodontitis, PAL < 1–2 > mm; Stage 4: Moderate periodontitis, PAL < 2–3 > mm; Stage 4: advanced periodontitis, PAL > 3 mm.

Based on the clinical findings, cats (*n* = 25) with periodontal indices of 3 and 4 (which were observed in at least three teeth) were selected as patients. These cats were deemed otherwise healthy according to clinical, laboratory, and ultrasonography findings. The control group (*n* = 25) included cats that did not show any signs of disease in the clinical examination, laboratory findings, and imaging studies, and were matched to the patient group based on breed, age, and gender. Age-matching of controls to subjects was conducted within a range of ± 1 years. Based on the clinical findings and laboratory evaluations, if cats showed signs of other disorders, including gastrointestinal, dermatological, cardiac, hepatic, renal disorders, or hyperthyroidism, they were excluded from the study.

### Hematology and biochemistry analysis

For hematology and biochemistry analysis, two milliliters of blood samples were collected through cephalic vein phlebotomy and immediately aliquoted into K2EDTA (Xinel, China) and clot activator tube (Vacutest®, Arzergrande, Italy). One portion of the serum was used for biochemical analysis, and a portion of the serum was stored for the evaluation of the antioxidant/oxidant status at -80 °C.

### Total antioxidant capacity (TAC)

The ferric reduction antioxidant power (FRAP) method, as described by Benzie and Strain [18], was used to measure the TAC. A commercial standard (Naxifer™) was employed for this purpose, and an ELISA reader (EMP, China) was utilized at a wavelength of 590 nm. The TAC results are reported in mmol Fe ^2+^/L.

### Total oxidant status (TOS), and oxidative stress Index (OSI)

A commercially available Natos™ Kit (Navand Salamat Co., Iran) was utilized to measure the level of TOS in serum. The detection method relied on the oxidation reaction facilitated by the presence of amplifying molecules in the reaction medium. The intensity of the resulting color, which was directly proportional to the total amount of oxidant molecules in the sample, was assessed using an ELISA reader (EMP, China). Hydrogen peroxide was used for calibration purposes, and the results were reported as equivalent liquid peroxide per liter (µmol Eq/L). Additionally, the OSI, calculated as the ratio of TOS to TAC, was determined for each group and expressed as µmol Eq /mmol Fe^2+^.

### Measurement of oxidized (GSSG) and reduced glutathione (GSH)

A commercially available NaglutTM GSSG assay Kit (Navand Salamat Co., Iran), and a commercially available NarGulTM GSH assay Kit (Navand Salamat Co., Iran) were utilized to measure the level of GSSG and GSH in serum. ELISA reader (EMP, China) was utilized at a wavelength of 420 nm. The GSSG and GSH results are reported in mmol/L and µmol/L, respectively. Additionally, the GSSG to GSH ratio, calculated as the ratio of GSSG to GSH by 100, was determined for each group and expressed as a percentage.

### Microbial culture

Subgingival plaque samples were obtained from all 25 healthy cats and 25 cats showing moderate to advanced periodontitis. All samples were collected before any dental treatment had been performed. Subgingival plaque samples were collected using sterile paper points, placed in sterile transport media and sent to the microbiology laboratory immediately after collection. To culture and isolate bacteria, sterile samples were inoculated onto sheep blood agar and MacConkey lactose agar plates and incubated at 37 °C for 24–48 h. Further evaluation was conducted through subculturing of the resulting growth onto blood agar plates and performing Gram staining, morphology, and colony characteristic analysis, as well as catalase and oxidase tests.

### Statistical method

All data were analyzed using the SPSS software version 22.0, and the Shapiro–Wilk test was used to assess the normal distribution. The unpaired t-test and Mann-Whitney U tests were used to compare differences between two independent groups. The Pearson and Spearman correlation coefficients were calculated to determine the strength and direction of the linear relationship between clinical, hematology and biochemistry findings and oxidative stress markers. The p-value threshold for significance was set at *p* < 0.05.

## Results

The signalment and clinical data of the apparently healthy cats and the cats with periodontitis, along with the mean ± SE of hematology, biochemistry, and oxidative factors are tabulated in Table 1. When the cats in the healthy control group were compared with the cats with moderate to severe periodontitis, the differences in the mean WBC, neutrophils, band cells, HCT, total protein, albumin, globulin, albumin to globulin ratio, and calcium concentrations were statistically significant (*p* < 0.05, Table 1). The evaluation of oxidative stress factors showed that the serum values of TAC in the control group were significantly higher than in the patient group (*p* < 0.05), and the TOS, OSI, GSSG, and GSSG.GSH ratio in the control group were significantly lower than in the patient group (*p* < 0.05, Table 1). However, the two studied groups did not have significant differences in GSH levels.

**Table 1:** Descriptive and analytical statistics of the study population (Mean ± SE)

Study Group	Healthy control (*n* = 25)	Periodontitis (*n* = 25)
**Signalment and Periodontal index**		
Age (year)	1.70 ± 0.28	2.33 ± 0.53
Sex	11 M/14F	13 M/12F
Periodontal Index ****	-	3.60 ± 0.08
Number of teeth with Periodontal index 3 and 4****	-	3.75 ± 0.21
**Hematology**		
WBC (cell*10^3^ /µL) **	6.61 ± 0.90	14.44 ± 1.52
Neutrophil (cell*10^3^ /µL) **	3.77 ± 0.77	8.83 ± 1.14
Band cell (cell*10^3^ /µL) *	0.02 ± 0.01	1.11 ± 0.02
Lymphocyte (cell*10^3^ /µL)	2.04 ± 0.292	2.50 ± 0.421
Eosinophils (cell*10^3^ /µL)	0.59 ± 0.09	0.67 ± 0.3
Monocyte (cell*10^3^ /µL)	0.12 ± 0.03	0.31 ± 0.07
Hct % **	34.42 ± 1.30	29.11 ± 1.10
MCV (fl.)	42.34 ± 0.75	44.49 ± 0.95
MCHC (gr/dl)	33.30 ± 0.57	32.80 ± 0.40
Plt (cell*103 /µL)	438.7 ± 73.11	358.9 ± 33.55
**Biochemistry**		
Total Protein (gr/dl) **	5.84 ± 0.25	7.58 ± 0.28
Albumin (gr/dl) ***	3.16 ± 0.05	2.69 ± 0.07
Globulin (gr/dl) **	2.75 ± 0.27	4.80 ± 0.30
Albumin to Globulin ratio ****	1.16 ± 0.13	0.58 ± 0.03
ALT (IU/L)	78.44 ± 9.65	77.39 ± 12.61
AST (IU/L)	46.93 ± 2.22	71.47 ± 14.30
ALP (IU/L)	138.4 ± 20.45	106 ± 14.30
GGT (IU/L)	3.85 ± 0.56	3.20 ± 0.53
Urea (mg/dl)	49.35 ± 2.95	57.33 ± 3.85
Creatinine (mg/dl)	1.07 ± 0.06	2.11 ± 0.43
Glucose (mg/dl)	103.2 ± 22.5	136.7 ± 87.8
Calcium (mg/dl) **	9.47 ± 0.16	8.10 ± 0.26
Phosphorous (mg/dl)	5.41 ± 0.29	5.10 ± 0.52
Total Bilirubin (mg/dl)	0.19 ± 0.04	0.56 ± 0.20
Triglyceride (mg/dl)	66.43 ± 7.86	76.09 ± 17.40
Cholesterol (mg/dl)	133.1 ± 9.40	143.2 ± 6.71
**Oxidative status**		
TAC (mmol Fe^2+^/L) ****	0.50 ± 0.03	0.20 ± 0.01
TOS (µmol Eq/L) ****	1.63 ± 0.15	4.07 ± 0.42
OSI (µmol Eq /mmol Fe^2+^) ****	3.39 ± 0.46	22.41 ± 2.30
GSH (µmol/L)	14.94 ± 0.90	14.35 ± 0.35
GSSG (mmol/L) *	0.15 ± 0.00	0.51 ± 0.10
GSSG/GSH ratio%*	1.10 ± 0.80	3.57 ± 0.71

* Significance levels are indicated by asterisks (*p < 0.05, **p < 0.01, ***p < 0.001, and ****p < 0.0001).

Table 2 displays the results of the correlation tests based on the Pearson and Spearman correlation coefficients. Only significant correlations are provided in this Table. In this evaluation, the correlation between hematological and biochemical profile changes with oxidative stress markers has been examined. Furthermore, the correlation between the PI and hematological and biochemical factors, as well as oxidative stress markers has been evaluated.

**Table 2:** Correlation results based on Pearson and Spearman correlation coefficient

	TAC	TOS	OSI	GSSG	GSSG. GSH ratio	PI
**WBC**	NS	*r*: +0.614	*r*: +0.421	*r*: +0.638	*r*: +0.647	*r*_*s*_: +0.567
		P: 0.0001	P: 0.018	P: 0.0001	0.0001	P: 0.001
**Band cell**	NS	*r*: +0.591	NS	*r*: +0.766	*r*: +0.701	*r*_*s*_: +0.582
		P: 0.0001		P: 0.0001	P: 0.0001	P: 0.0001
**PMN**	NS	*r*: +0.668	NS	*r*: +0.645	*r*: +0.672	*r*_*s*_: +0.630
		P: 0.0001		P:0.0001	P: 0.0001	P: 0.0001
**Monocyte**	NS	*r*: +0.344	NS	*r*: +0.424	NS	NS
		P: 0.043		P: 0.008		
**Hct**	NS	*r*: -0.451	NS	*r*: -0.413	NS	*r*_*s*_: -0.361
		P:0.007		P: 0.01		P: 0.03
**Tp**	*r*: -0.493 P: 0.003	*r*: +720	r: + 0.601 P: 0.0001	*r*: +0.661	*r*: +556 P:001	*r*_*s*_: +0.700
		P:0.0001		P: 0.0001		P: 0.0001
**Albumin**	PC: +0.413	*r*: -0.631	*r*: -0.446	*r*: -0.471	*r*: -0.389	*r*_*s*_: -0.664
	P: 0.015	P:0.00001	P: 0.008	P: 0.003	P: 0.025	P: 0.0001
**Globulin**	NS	NS	NS	NS	NS	*r*_*s*_: +0.389
						P: 0.019
**A.G ratio**	NS	*r*: -0.394	NS	NS	NS	*r*_*s*_: -0.509
		P: 0.019				P: 0.002
**Calcium**	PC: +0.482	NS		NS	NS	*r*_*s*_: -0.587
	P: 0.004					P: 0.0001
**Urea**	NS	NS	NS	NS	NS	*r*_*s*_: +0.327
						P: 0.048
**PI**	*r*_*s*_: -0.693	*r*_*s*_: +0.760	*r*_*s*_: +0.647	*r*_*s*_: +0.822	*r*_*s*_: +0.760	1
	P: 0.0001	P: 0.0001	P: 0.0001	P: 0.0001	P: 0.0001	

Note: The table displays the Pearson correlation coefficients (r-values) and Spearman correlation coefficients (*r*_*s*_ -values) for various pairs of variables alongside their corresponding p-values. Positive values indicate a positive correlation, negative values indicate a negative correlation

In summary, the results of the correlation test indicated that among the antioxidant factors examined, TAC showed a significant positive correlation with albumin and calcium, while serum GSH did not show a significant correlation with any factor. On the other hand, each of the oxidant factors including TOS, OSI, GSSG, and GSSG.GSH ratio exhibited at least one significant correlation with the hematological and biochemical factors.

Among the hematological factors, the leukocyte count demonstrated the highest correlation with oxidative stress factors, followed by the total band cell count and neutrophil count. Among the biochemical factors, serum albumin showed the highest correlation with oxidative stress factors. Additionally, the results showed that the PI had a significant positive correlation with all evaluated oxidant factors and a significant negative correlation with TAC. Furthermore, the PI had a significant correlation with some hematological and biochemical factors as well.

### Microbial culture results

The results of bacterial culture are shown in Table 3. A total of 5 bacterial species and 31 species-specific colonies were isolated in healthy cats. At least one and a maximum of 2 colonies were isolated from each cat. Pseudomonas aeruginosa was seen only in a healthy cat. A total of 9 bacterial species and 96 species-specific colonies were isolated in cats with periodontitis. At least two and a maximum of 5 colonies were isolated from each patient. *Staphylococcus chromogenes*, *Bacillus spp., Trueperella pyogenes, Streptococcus canis*, and *Corynebacterium spp.*, were seen only in the patient group.

**Table 3:** Gingival microbial culture results in two groups of healthy cats and cats with moderate to severe periodontitis

	Total number of times each bacterium was isolated	
	Healthy control (*n* = 25)	Periodontitis (*n* = 25)
*Pasteurella multocida*	12 (39%)	25 (26%)
*Streptococcus α-haemolytic*	10 (32%)	22 (23%)
*Staphylococcus epidermidis*	5 (16%)	6 (6%)
*Staphylococcus pseudintermedius*	3 (10%)	21 (22%)
*Staphylococcus chromogenes*	0	6 (6%)
*Bacillus spp*	0	3 (3.5%)
*Trueperella pyogenes*	0	4 (4%)
*Streptococcus canis*	0	6 (6%)
*Corynebacterium spp*	0	3 (3.5%)
*Pseudomonas aeruginosa*	1 (3%)	0
Sum	31	96

## Discussion

Periodontal diseases comprise a wide range of inflammatory conditions and are the result of inflammation and infection of the gingiva and bones around the teeth. In the early stages of the disease called gingivitis, the gums become swollen, red, fragile, and prone to bleeding. In its more severe manifestation known as periodontitis, the gums can recede and separate from the teeth, bone loss may occur, and teeth may become unstable or even dislodge [19, 20]. Gingivitis and periodontitis are the most commonly diagnosed health problems in cats, associated with regional disorder and pain, and as a consequence, difficulty feeding, weakness and also systemic effects, and multi-organ failure [1]. Previous studies in humans have shown there is a strong association of periodontal diseases with several systemic disorders [21]. Despite this, very little is known about the association and pathogenicity of organ disorders with periodontal diseases in cats. Intending to address this knowledge gap, the main aim of our study was to illustrate the influence of moderate to severe periodontitis on oxidative stress parameters by investigating serum TAC, TOS, OSI, GSH, GSSG, and GSSG.GSH ratio values in cats. In the current study, the two studied groups were not significantly different in terms of age and gender, and the average age of all cats was less than three years. The reason for choosing young cats was to prevent as much as possible the occurrence of diseases that increase with age. Also, if any clinical and laboratory evidence of diseases other than periodontitis was observed, the patient was excluded from the study.

In a recent study on a colony of cats, the results showed that an average of 12% of the teeth in cats show signs of periodontal attachment loss [17]. In the current study, to create similar conditions, cats were selected that showed signs of attachment loss in at least 3 teeth, which is equivalent to 10% of all permanent teeth.

Comparing the hematological and biochemical results between the control and patient groups showed the presence of significant inflammatory conditions in cats with moderate to advanced periodontitis. In affected cats, leukocytes, neutrophils, band cell counts, total protein and globulin values were higher, and hematocrit, serum albumin, albumin to globulin ratio, and calcium levels were lower, significantly.

Periodontitis, similar to other chronic inflammatory diseases, may lead to anemia of chronic disease (ACD). In this condition, due to the production of inflammatory cytokines including TNF and IL-1, both the production of erythropoietin and the bone marrow^’^s response to erythropoietin are reduced [7, 22]. This type of anemia is the main cause of anemia in both human and veterinary medicine and in dogs and cats can occur within 1 to 2 weeks or faster [23].

In affected cats, albumin probably decreased as a negative acute-phase protein, and subsequently, the decrease in albumin caused a significant decrease in total calcium. Additionally, the results of correlation tests showed that plasma albumin level compared to other evaluated inflammatory factors such as globulin, A/G ratio and CBC findings, had more significant correlations with stress oxidative factors. These results indicated that albumin had a significant positive correlation with TAC, and significant negative correlations with TOS, OSI, GSSG, GSSG.GSH ratio, and PI. Albumin, as the most relevant negative acute-phase protein in cats, exhibits decreased blood concentration during inflammation. These decreased can be attributed to a shift in amino acid utilization towards the synthesis of positive acute-phase proteins [24, 25].

Previous studies in man showed there is a correlation between periodontal disease and systemic markers of inflammation [4]. Patients with chronic periodontitis have higher circulating leucocytes, acute-phase proteins, coagulation factors, albumin, serum C-reactive protein, and cytokines such as IFN-γ, TNF-α, IL-1β, IL-2, and IL-6, IgG1 and IgG2 [3, 4]. Similar studies in cats with periodontal diseases showed that advanced dental disease is associated with measurable systemic changes. A study of cats with periodontal disease examined the correlation between several routine clinicopathological variables and the severity of the disease [7]. In that study, the authors found that total globulins, ALT, and IgG were positively correlated, and albumin, hemoglobin, hematocrit, and AST were negatively correlated with the severity of the disease. Furthermore, the authors stated that the inflammatory markers improved following appropriate dental treatment. The logical conclusion is that periodontitis-associated local inflammation can have remarkable systemic manifestations.

Gingivitis and periodontitis as local inflammatory diseases are usually initiated by bacterial infection and subsequently progressed by aberrant host response [26]. In these situations, the excessive production and release of reactive oxygen, which are overproduced mostly by hyperactive neutrophils, has been implicated in the development of destructive local diseases. Previous studies in man have shown that enhanced local and systemic oxidative stress in patients with periodontitis can be a significant mechanism linking periodontitis to several systemic disorders [10]. Oxidative stress can cause functional alteration of several enzymes, lipid peroxidation, protein and DNA damage, and aberrations in gene expression, and therefore can play a key role in the etiopathogenesis of several systemic chronic diseases such as diabetes, cardiovascular diseases, neurodegenerative diseases, and cancer [10, 27].

Therefore, the presence of oxidative stress in cats with periodontitis can be a contributing factor to their susceptibility to certain types of other disorders. Previous studies have shown that cats with periodontitis are prone to renal disease [8].

The results of the correlation tests showed that among the clinical, biochemical, and hematological factors examined, oxidative stress factors had the highest correlation with PI, albumin, leukocytes, band cells, and neutrophils. Furthermore, the results indicated that among the types of oxidant and antioxidant factors, the oxidant factors showed a greater correlation with various clinical and laboratory indicators. Previous studies have showed a range of increased to decreased TAC may be observed in the patients with periodontitis [13]. In these patients an initial increase of TAC may be seen in early stages of the disease, and the authors attributing this to a protective response to oxidative stress. This initial responsive reaction may decrease with the progression and duration of the disease [13].

However, it appears that oxidant factors are generally increasing in these patients. Therefore, oxidant factors such as TOS and OSI, compared to antioxidant factors, may better indicate the presence of oxidative stress conditions in patients with periodontitis. Similarly, some studies have shown the superior value of oxidative parameters over antioxidant parameters in evaluating oxidative stress in patients with periodontitis [10].

In the current study, despite evidence of oxidative stress in patients, no significant difference was observed in serum GSH levels between the control and patient groups. There are some possible explanations for these results: (1) The serum and extracellular levels of glutathione are much lower compared to its intracellular levels [28], as it is found inside the cells in millimolar concentrations, while extracellularly they are found in micromolar. So, the extracellular concentration of GSH may not be a sensitive indicator of GSH changes. So, the evaluation of GSH in tissue samples, compared to serum, may be preferable for demonstrating GSH changes in patients. However, GSSG is found mainly extracellularly [28] and serum GSSG levels are much higher than GSH, and its changes can be tracked with greater sensitivity in the serum. (2) The use of more sensitive measurement methods such as HPLC instead of biochemical methods may increase the sensitivity of serum glutathione measurement [29]. (3) Previous studies have shown that conditions such as oxidative stress, inflammation, and cancer can induce GSH synthesis in the body, so GSH levels may be raised, attributing to a protective and adaptive response to oxidative stress [30–32]. In the present study, the presence of severe inflammatory conditions in the cats may have likely led to increased GSH synthesis, therefore, a significant decrease has not been observed in the patients. However, considering the presence of oxidative conditions in patients, the level of GSSG and the ratio of GSSG to GSH were significantly higher in patients compared to the control group.

The correlation test results demonstrated that PI is significantly correlated not only with various oxidative stress factors but also with several hematological and biochemical factors. The correlation of PI with urea may potentially indicate the presence of pre-renal azotemia in cats affected by periodontitis. In periodontitis conditions, due to the fragility and inflammation of the gum tissues, there is a higher likelihood of oral bleeding, which can lead to pre-renal azotemia [33]. It should be noted that patients showing signs of renal failure, heart failure or other disorders were excluded from the current study.

Periodontal diseases is expected to increase both the number and range of bacterial species present in the mouth [7]. Similarly, in the current study, the results of microbial culture showed that the number of bacterial species isolated from the gums of cats with periodontitis was 2 to 5 species, while in healthy cats, one to two bacterial species were isolated from each cat. Also, the diversity of bacterial species was higher in sick cats. Thus, from a total of 25 sick cats, 9 bacterial species were isolated in the form of 96 colonies, but from healthy cats, 5 species were isolated in the form of 31 colonies. Although this study cannot rule in any effect of different species of bacterium on the periodontal disease pathogenicity, microbial culture results are remarkably different between healthy control cats and cats with periodontitis. The most common bacterial species isolated from healthy and sick cats was *Pasteurella multocida*, whose relative prevalence was lower in sick cats compared to healthy cats. Mallonee et al., have shown *Pasteurella multocida* was isolated from most samples and appeared to decrease in numbers with increasing periodontal disease [34]. Furthermore, in the present study *Staphylococcus chromogenes, Bacillus spp., Trueperella pyogenes, Streptococcus canis, and Corynebacterium spp.*, were seen only in cats with periodontitis.

In periodontitis treatment, the choice of therapeutic interventions depends on various factors such as the specific dental condition, the extent of damage, and the overall health of the patient. Plaque and tartar removal along with the use of topical antibiotics may be sufficient for patients with mild to moderate periodontitis. However, in cases of severe periodontitis, it may be unavoidable to use systemic antibiotics or perform tooth extraction [20].

Human studies have demonstrated that not only periodontal therapy reduces the levels of oxidative stress biomarkers, but the prescription of antioxidants itself is effective in the process of treating periodontitis [35, 36].

Prevention of periodontitis in cats is essential for maintaining their oral health. Regular dental examinations, dental cleanings, teeth brushing, and dental diets and treats are recommended important measures for preventing periodontitis in cats [20].

This study has three main limitations. The first limitation was the relatively low number of cats included in the study. Each group consisted of only 25 cats, which may have limited the statistical power and generalizability of our findings. Future studies with larger sample sizes could provide more robust results and allow for better extrapolation to the general cat population. The second limitation was the relatively low number of stress oxidative factors evaluated in this study. While we assessed a set of well-established stress oxidative markers, the inclusion of additional factors may have provided a more comprehensive understanding of the oxidative stress status in cats with periodontitis. Future studies should consider examining a wider range of stress oxidative markers to obtain a more complete picture of this biological pathway’s involvement. Furthermore, a noteworthy limitation is that we did not monitor the cats after periodontal treatment. Assessing the stress oxidative factors before and after treatment would have allowed us to observe any changes in the oxidative stress status following intervention. This information could have provided valuable insights into the impact of periodontal treatment on oxidative stress levels in cats. Despite these limitations, our study adds valuable information about the correlation of periodontitis and stress oxidative status in cats. It highlights in cats with moderate to severe periodontitis, there is a higher likelihood of the presence of stress oxidative. The present study’s results indicate that the assessment of PI in cats can be a potential predictive factor of oxidative stress status in patients.

## Conclusion

In the present study, the results showed moderate to advanced periodontitis in cats can exacerbate and promote the progression of systemic oxidative stress in cats. In addition, data analysis suggests that evaluating the oxidant factors such as TOS and OSI, in comparison to the antioxidant factors, may better indicate the presence of oxidative stress conditions in cats with periodontitis. These data now provide a mechanistic link between periodontitis and the regional and systemic effects of the disease in cats, and could lead to potential therapies for oxidative stress-induced tissue damage.

## Data Availability

The data used and/or analyzed during the current study are available from the corresponding author upon reasonable request.

## Abbreviations

TAC: Total antioxidant capacity
TOS: Total oxidant status
GSH: Reduced glutathione
GSSG: Oxidized glutathione
OSI: Oxidative stress index
PP: Periodontal probing depth
R: Gingival recession
PAL: Periodontal attachment loss
PI: Periodontal Index
ARRIVE: Animal Research: Reporting of In Vivo Experiments
EDTA: Ethylenediaminetetraacetic acid
EMP: Emperor
FRAP: Ferric reduction antioxidant power
ELISA: Enzyme-linked immunosorbent assay
SPSS: Statistical Package for the Social Sciences
ACD: Anemia of chronic disease
TNF: Tumor necrosis factor
IL: Interleukin
IFN: Interferon
WBC: White blood cells
Hct: Hematocrit
MCV: Mean corpuscular volume
MCHC: Mean corpuscular hemoglobin concentration
Plt: Platelet
ALT: Alanine transaminase
AST: Aspartate transferase
ALP: Alkaline phosphatase
GGT: Gamma-glutamyl transferase
Tp: Total protein
A.G ratio: Albumin to globulin ratio
SE: Standard error
NS: Non significant
